# A Case of Poisoning from Opium, Successfully Treated by Electro Magnetism

**Published:** 1851-07

**Authors:** J. B. Biddle


### ARTICLE IV.

### *A Case of Poisoning from Opium, successfully treated by Electro Magnetism.* By J. B. Biddle, M. D.

The following case illustrates, I think, very strikingly, the value of the electro-magnetic current as a means of relieving the coma produced by narcotic poisoning.

At about half-past twelve o'clock of the night of the twenty-eighth of April last, I was called to visit a woman, described by the messenger as being in a fit. No history or explanation of the case could be obtained, except that the patient had gone out at about half-past seven o'clock to get something at an apothecary's for a cramp colic; that she had upon her return home eaten her supper as usual, then gone to bed, soon fallen into deep sleep, and finally, at about midnight, from her unusual respiration and the impossibility of rousing her, excited the alarm of her husband and family.

I found her in a state of profound torpor; her breathing extremely slow and interrupted, stertorous and gasping, with spasm of the throat, lividity of the countenance, inability to swallow, utter insensibility to the most violent agitation, pupil contracted to the size of a pin's head, pulse scarcely perceptible at the wrist—in short, all the symptoms of an advanced stage of asphyxia. That it was a case of narcotic poisoning, rapidly approaching a fatal termination, was, I thought, evident, and I at once so expressed myself—the family, however, still professing themselves unable to explain or account for it.

Acting, however, upon this opinion, I obtained the assistance of my friend, Dr. Goddard, who lives in the neighborhood, and the use of his electro-magnetic apparatus; and, the doctor coinciding in my view of the case, we derermined, although with no very strong hope of saving the woman's life, to resort to this agent. An attempt was made to introduce the stomach tube, but was unsuccessful, owing to spasm of the pharynx, and its introduction could have been of no service, as, at the lapse of more than five hours, the poison must have been altogether absorbed from the stomach.

The electro-magnetic machine employed consists of two coils rotating between the poles of two horse-shoe magnets—

an unusually large and powerful instrument, producing a rapid succession of violent shocks. One pole was applied to the nape of the neck, the other to the pit of the stomach. For about two minutes after the battery was started no effect was produced. The patient then began to make convnlsive efforts with her hands, as if to put away something annoying her, and, in perhaps half a minute more, she opened her eyes, with a ghastly stare. The battery being still kept in action, she rose up in bed, and was able to mutter some indistinct answer to questions put her.

Upon withdrawing the electric current, the woman immediately sank back into the state of torpor in which I had found her. But, us soon as it was renewed, artificial vitality was again restored. But when the current was a second time stopped, after about the same period of application as at first, the woman continued for some two or three minutes awake, gradually, however, relapsing into coma. After each application of the battery, the interval of consciousness became longer, and, at the end of two hours, she remained roused for a full half hour, in which she was able to let us know what she had taken.

It appears that she had bought "three cents" worth of laudanum, and, never having taken it before, she supposed it was a proper dose, and swallowed it all. It amounted, as she said, to some three teaspoofulls—probably two fluid drachms, as this is, I believe, the quantity usually sold for that price. I think it probable that she was also previously somewhat under the influence of whisky, as we detected it on her breath, and this must have increased the narcotic effect of the laudanum.

We now gave her some volatile alkali, and strong coffee, but they were not long retained. After half an hour's consciousness, stupor slowly crept on again, and a further resort was had to the battery, which was followed with rapid, and, as it proved, a final revival.

The patient now got up, walked about, conversed clearly, was able to keep some coffee on her stomach, and it was apparent that she had at last struggled through the effects of the narcotic. Some disposition to somnolence remained, but this was easily overcome without recourse to the battery. I remained with her till half-past four—an hour and a half from the last application of the electricity, and then left her in charge of her friends, directing them not to suffer her to sleep till I saw her again.

Between eight and nine I found her very comfortable and completely awake, although begging hard to be allowed a nap. Three or four hours natural sleep now took place, and left her completely recovered.

It may be worth mentioning, that in the successive application of the poles of the battery, while one was kept constantly to the nape of the neck, the other was placed indifferently at the pit of the stomach, the arm-pit, and in the hand; and the effect did not appear to vary.

Since drawing up the notes of this case, upon mentioning it to my friend, Dr. Mutter, I found that he had lately resorted to electro-magnetism with success under similar circumstances; and he kindly offered the history of his case for publication with the foregoing:

*May* 14*th*, 1851.

DEAR DOCTOR:—In accordance with your request, I send a brief outline of the case of "poisoning with opium," to which I referred in our interview the other day.

Last spring, my colleague, Prof. Pancoast, and myself, were summoned about 11 o'clock, P. M., to visit a young gentleman residing at the corner of Ninth and Market streets. On our arrival, we found that a large quantity of laudanum had been swallowed accidentally, and although strong and very appropriate means had been immediately taken by several medical students who lodged in the same house, no impression seemed to be made upon the influence of the drug. All the evidences of rapidly approaching death were manifest, and as all other measures had been unsuccessfully employed, we determined to employ *electro-magnetism*. An instrument was accordingly obtained, one pole placed upon the nape of the neck, and the other over the epigastrium. Almost on the instant, the muscles of respiration were violently agitated, and the patient sprang up in bed, opened his eyes, and answered questions. The pain in a few moments was so severe, that we were obliged to change the position of the machine. Keeping one steadily applied to the back of the neck, the other was made to touch different points of the thorax, throat, abdomen and upper extremities. The *burning* sensation occasioned by the fluid, was almost intolerable, causing the patient to complain loudly, and effectually preventing any return to the lethargy from which he had so happily been aroused. We deemed it most prudent to continue our efforts, even after the patient was fully restored to consciousness, but I think that not more than *an hour* elapsed between the first application of the remedy and the complete relief of our young friend.

Yours truly, THOS. D. MUTTER.

*Dr. Biddle.*

